# Evaluating Thermal Infrared Drone Flight Parameters on Spider Monkey Detection in Tropical Forests

**DOI:** 10.3390/s24175659

**Published:** 2024-08-30

**Authors:** Eduardo José Pinel-Ramos, Filippo Aureli, Serge Wich, Steven Longmore, Denise Spaan

**Affiliations:** 1 Instituto de Neuroetología, Universidad Veracruzana, Av. Dr. Luis Castelazo Ayala, Xalapa 91190, Veracruz, Mexico; faureli@uv.mx; 2 ConMonoMaya, A.C., Km 5.4 Carretera Chemax-Coba, Chemax 97770, Yucatán, Mexico; 3 School of Biological and Environmental Sciences, Liverpool John Moores University, James Parsons Building, Byrom Street, Liverpool L3 3AF, UK; s.a.wich@ljmu.ac.uk; 4 Astrophysics Research Institute, Liverpool John Moores University, 146 Brownlow Hill, Liverpool L3 5RF, UK; s.n.longmore@ljmu.ac.uk

**Keywords:** unoccupied aerial vehicles, population monitoring, *Ateles*, primates, flight speed, flight height, drone camera angle, Yucatan Peninsula

## Abstract

Geoffroy’s spider monkeys, an endangered, fast-moving arboreal primate species with a large home range and a high degree of fission–fusion dynamics, are challenging to survey in their natural habitats. Our objective was to evaluate how different flight parameters affect the detectability of spider monkeys in videos recorded by a drone equipped with a thermal infrared camera and examine the level of agreement between coders. We used generalized linear mixed models to evaluate the impact of flight speed (2, 4, 6 m/s), flight height (40, 50 m above ground level), and camera angle (−45°, −90°) on spider monkey counts in a closed-canopy forest in the Yucatan Peninsula, Mexico. Our results indicate that none of the three flight parameters affected the number of detected spider monkeys. Agreement between coders was “substantial” (Fleiss’ kappa coefficient = 0.61–0.80) in most cases for high thermal-contrast zones. Our study contributes to the development of standardized flight protocols, which are essential to obtain accurate data on the presence and abundance of wild populations. Based on our results, we recommend performing drone surveys for spider monkeys and other medium-sized arboreal mammals with a small commercial drone at a 4 m/s speed, 15 m above canopy height, and with a −90° camera angle. However, these recommendations may vary depending on the size and noise level produced by the drone model.

## 1. Introduction

Drones have become an increasingly popular tool for wildlife monitoring because this technology is becoming more affordable, easier to obtain [1,2], and can offer several advantages over traditional monitoring methods [3]. One of the key advantages is their ability to survey large areas rapidly and efficiently, providing detailed aerial imagery from which population measures such as animal distribution and abundance (number of individuals or groups in an area) can be calculated. Such calculations rely on information on the presence of the animal of interest in the area (distribution and occupancy) or the number of individuals present in the area (abundance, population density). In turn, these measures are influenced by the detectability of the animal of interest [4,5].

Detectability refers to the probability of sighting an individual animal during a survey when the animal is present in the surveyed area [6,7]. Detectability can be influenced by different factors such as habitat type, species abundance, social structure, animal behavior, weather conditions, and survey methods [8,9,10] and can significantly affect population measures [6]. The latter occurs because the factors affecting detectability can lead to false negatives and false positives. False negatives occur when individuals that are present are not detected [8], and therefore lead to the underestimation of population parameters. False positives occur when individuals of another species or other elements in the environment are recorded as the species of interest [8], and lead to the overestimation of population parameters. It is therefore crucial to understand the factors that affect detectability so that they can be accounted for statistically, e.g., using hierarchical modeling (e.g., occupancy modelling, N-mixture modeling), and surveys can be designed to obtain accurate population estimates [11].

Some of the main factors influencing animal detectability with drones are the type of sensor employed and the flight parameters used to program the surveys. Among the most commonly used sensors are acoustic, image, and thermal sensors [12]. Acoustic sensors (sound recorders) can detect sounds emitted by animals, including their vocalizations and mating calls [13]. Image sensors (red–green–blue RGB visual spectrum cameras) can detect the presence of animals or their signs (e.g., great ape nests [14,15]) by capturing high-resolution images or videos [16,17,18,19,20]. Thermal infrared (TIR) cameras can detect the presence of animals by detecting the temperature difference between the animal and its environment [21,22,23,24,25]. The use of drones with TIR cameras (hereafter TIR drones) allows for the detection of animals in low light conditions or at night, which may make it possible to obtain data on cryptic or nocturnal species, or species with ecological characteristics that make it very difficult to obtain accurate and up-to-date information on their distribution, such as arboreal non-volant mammals [23,24,26]. In recent years, the number of studies aimed at determining the distribution or abundance of arboreal mammals using TIR drones has increased considerably (howler monkeys *Alouatta palliata* [21,25]; koalas *Phascolarctos cinereus* [27,28]; orangutans *Pongo pygmaeus* [29]; long-tailed macaques *Macaca fascicularis* [30]; spider monkeys *Ateles geoffroyi* [21,23,25]; kinkajous *Potos flavus* [25]; northern muriqui *Brachyteles hypoxanthus* [31]; greater gliders *Petauroides volans* [32]; Hainan gibbon *Nomascus hainanus*: [24,33]; and Lumholtz’s tree kangaroo *Dendrolagus lumholtzi* [34]), but knowledge gaps still remain regarding the flight parameters that maximize the detectability of species in different habitat types.

When using TIR drones, it is important to consider several factors that can affect arboreal mammal detection. For example, the time of day at which flights are conducted can be crucial, because if they are conducted during daylight hours with high solar radiation, environmental elements, such as leaves and branches, absorb that radiation and can have temperatures similar to those of arboreal mammals [21]. This situation can make it challenging to differentiate individuals from their environment, potentially leading to false negatives or false positives. Therefore, the best time to fly drones with TIR cameras is at night or around dawn and dusk [21,25,35], although flying during the day has been successful under heavy cloud cover (Fabiano de Melo, personal communication) and in areas where the body temperature of endotherm animal species is easily differentiated from the temperature of the other elements of the environment [36]. The time of the day to fly drones may be highly variable in mountain environments. Forests located on different sides of a mountain receive sunlight at different times of the day, lengthening the possibility for early-morning TIR drone flights (if sunlight is received later in the morning) or early-evening flights (if the sun sets early in the day). The presence of human infrastructure in the environment (e.g., roads or houses) also absorbs solar radiation and retains it for a prolonged period of time [37], which may hinder the detection of arboreal mammals. Surveying arboreal mammals in heterogenous habitats (i.e., where there is a combination of natural and anthropogenic elements, such as roads, houses, and other infrastructure, or a variety of natural elements that differ in thermal contrast such as rocky outcrops, rupestrian fields, or grasslands) can result in differences in detectability between zones with high thermal contrast (e.g., forested areas where the temperature difference between the animals and environmental elements is high) and zones with low thermal contrast (e.g., areas with anthropogenic infrastructure with high temperatures that limit the detectability of the animals). Therefore, knowing the conditions of the study site in advance can help to plan flight survey designs that overcome these limitations.

Previous studies show that flying TIR drones at night maximizes the temperature difference between the study species and their environment, and thereby enhances their detectability [21,25,38]. This increased contrast between the temperature of the study species and the environment can also include diurnal species that spend time in parts of the environment where they can be detected. To date, little research has been conducted on other factors that may affect arboreal mammal detectability in TIR drone footage. Flight speed influences the overall survey area that can be covered in a single flight. For instance, when flying slower, the battery may last longer but the area covered is smaller than when flying faster. Although more area can be covered when flying faster, this causes a decrease in the level of detail in the TIR drone footage, making it more difficult to differentiate individuals from other elements of the environment, and some individuals may even remain undetected [25]. This is because flight speed influences the accuracy with which different arboreal mammal species can be detected and the individuals taxonomically classified [21,25]. Although flying faster does not impact the number of detections of group living species, it negatively impacts the number of detections of solitary species [25], because it is easier to detect a group of individuals than a single individual in TIR drone footage. Although previous studies have shown that flight speed influences the level of detail that can be observed in TIR drone footage [21,25], it is not yet clear what flight speed balances fast sampling and maintaining sufficient detail to achieve a species level taxonomic determination of the sighted individuals.

The height at which the drone is flown is another factor that can influence the detectability of the study species [21,39,40,41,42]. The appropriate flight height depends on the size of the animals and location, as well as the environmental conditions and objectives of the study [38]. The topography of the terrain and the height of the trees are important factors to take into account when deciding the flight height. For example, in areas where the topography is irregular, presenting a high variation in the elevation, e.g., mountainous areas, and/or where trees differ largely in height, it is necessary to consider such variation when deciding the flight height to avoid accidents that may endanger the physical integrity of wildlife, humans, and the drone [43]. Flying lower above the ground increases image resolution, improving the differentiation of the species of interest from other detected species, and results in a higher detectability [21,38,44]. For TIR drones, it is recommended that the individuals appear at least ten pixels in size in the image for the camera to perform the temperature measurement [38]. To achieve this minimum pixel size, the height at which the drone is flown must reflect the size of the species of interest. For smaller species, the drone needs to be flown lower than for larger species.

The effects of flight height on mammal detectability have been tested for different species with drones equipped with RGB visual cameras, such as kangaroos (*Macropus giganteus* [39]) and hippopotamus (*Hippopotamus amphibius* [40]), and with TIR drones (European hare *Lepus europaeus* [41]). Although flying the drone higher results in a decrease in image resolution (and therefore detectability), a larger sampling area can be covered in a single flight, and the potential disturbance to the study species is lesser than when flying at lower heights [39,42]. However, more information is needed as to the maximum height that can be flown before detectability is too low. It is therefore necessary to balance achieving detection of the species without affecting the animals or altering their natural behavior. Although appropriate flight height has been evaluated for some species, it must be evaluated at the species level, as different species have different characteristics (e.g., body size and levels of tolerance to disturbance). The appropriate flight height should therefore ensure a high image resolution, animal wellbeing, and the optimization of flight time and battery life [21].

Another critical factor to consider when monitoring arboreal mammals using TIR drones is the camera angle, although it is one of the least evaluated factors [45]. The angle at which the camera is positioned can affect both the detectability of the animal and the amount of area that can be covered during a single flight [38]. However, it must be noted that choosing a fixed camera angle also depends on the terrain where one is flying. At a −90° angle, the camera is pointed downwards, creating a flat image of the tree canopy. Individuals that are located under the leaves of the canopy trees may therefore go undetected. This is because TIR sensors detect the temperatures of the surface elements of the forest (i.e., the very top of the canopy) and cannot penetrate to the lower or middle parts of the canopy in the absence of canopy gaps [38]. A −90° angle increases the probability of “poacher” detection in canopy gaps when using a TIR camera compared to a −45° angle [35]. At a −90° angle, the leaves and small branches at the top of the canopy make up most of the background elements, but leaves do not absorb and retain as much heat as tree trunks, which make up a greater proportion of the background elements with a −45° angle. As such, the contrast between the “poachers” and the background elements is greater at a −90° angle, facilitating their detection [35].

With a camera angle at −45°, individuals stay in the drone’s field of view for a greater amount of time, which in principle may facilitate their detection [38]. However, when using a −45° angle, the apparent size of any object in the field of view is distorted [38], which may make it difficult to determine whether particular objects are individuals of a given species. At the same time, this distortion of the field of view results in a larger sampling area being covered at a −45° angle than at a −90° angle, generating an image with a narrower area at the bottom of the field of view and a wider area at the top. Despite its importance, research on identifying the optimal camera angle for species detectability with TIR drones is limited. Therefore, evaluation of this flight parameter is crucial for the effective implementation of future drone surveys.

In the case of primates, several studies have carried out TIR drone surveys using different flight heights, speeds, and camera angles (Table 1). Most TIR drone surveys of primate populations used a −90° angle configuration, while the speed and flight height were highly variable (Table 1). However, only a few studies have evaluated how these flight parameters influence species detectability [25,29,30,42,46].

Geoffroy’s spider monkeys (*Ateles geoffroyi*) are diurnal, arboreal, fast-moving primates with a high degree of fission–fusion dynamics [55], making it difficult to obtain accurate population estimates with traditional methods such as line-transect surveys [20,23,56]. However, information on the distribution and abundance of their populations is vital because the species is listed as Endangered on the IUCN Red List of Threatened Species [57] and has been listed as one of the world’s 25 most endangered primate species [58]. Constant monitoring of their populations is therefore needed to identify population changes over time and create timely and targeted conservation programs [58]. To date, TIR drones have successfully detected and counted spider monkeys [21,23,25]. The influence of TIR drone flight speed on the detectability of spider monkeys and other primate species was evaluated in Costa Rica [25]. Detectability remained stable for primate species as a whole across flight speeds [25], suggesting that flights can be performed at high speeds, thereby covering larger sampling areas in a single flight, saving time and drone battery. However, it is unknown whether this result only applies to the entire primate community at the site or whether it also applies to each single species. While these studies have helped to understand the feasibility of using TIR drones for spider monkey monitoring, information on the flight parameters that ensure greater spider monkey detectability is lacking. For example, the appropriate flight height and camera angle needed to monitor spider monkey populations that balance the overall area covered during the flight (and therefore flight time and drone battery life), the species wellbeing, and the level of detail needed to clearly determine whether the detected animals are spider monkeys (therefore providing accurate abundance estimates), remain unclear.

Another important aspect to take into account is the reliability of the results obtained from manually coding TIR drone footage (i.e., manual review of videos to detect and count individuals of the species of interest). Manual coding of TIR imagery remains common practice for many research groups [25,41,42]. Although there has been an increase in the development of algorithms for the automatic detection of wildlife species with TIR cameras [19,59,60], not everyone has access to these, and the feasibility of using automatic detection algorithms in complex environments (i.e., with irregular topography, dense canopy cover, and the presence of elements that may complicate the detection of individuals) still remains poorly understood [59]. Inter-coder agreement is fundamental for result validity in population-based studies [61] and ensures the consistency and reliability of individual animal detection and species identification in TIR drone footage.

The first aim of our study was to evaluate how flight parameters such as flight speed, flight height, and camera angle influence the number of spider monkeys detected in videos recorded during flights with a TIR drone. We expect that flight parameters that allow for a more detailed observation of individual spider monkeys lead to a greater number of individuals to be detected in the videos. We therefore predict a higher count of spider monkeys in videos recorded when the drone is flown at lower flight speed, lower height, and with the camera positioned at a −90° angle than when the drone is flown with other parameters. The second aim was to compare the level of agreement between the main coder and two additional coders with varying levels of experience on the number of spider monkeys detected in the videos depending on flight parameter combinations and thermal contrast zones. We predict a higher inter-coder agreement in high thermal contrast zones in the videos recorded at lower speed, lower heights, and with the camera angle at −90°.

## 2. Materials and Methods

### 2.1. Study Site

**Figure 1:**
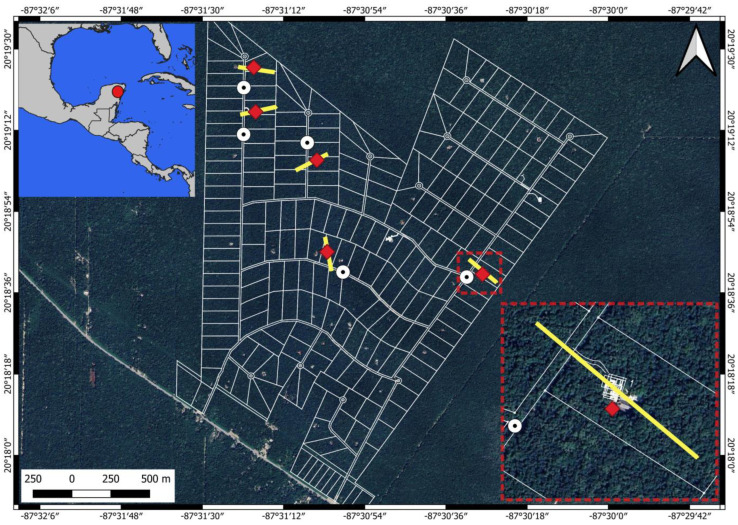
Map of Los Arboles Tulum, Tulum, Mexico, with 2 ha lots (white lines) showing the drone take-off and landing points (white dots with a black center) and flight routes (yellow lines) over five spider monkey sleeping sites (red squares) where we tested the effect of three flight parameters on spider monkey detectability.

The study was conducted in Los Arboles Tulum (20°17′50′′ N, 87°30′59′′ W), located in the municipality of Tulum, Quintana Roo, Mexico (Figure 1). Los Arboles Tulum (hereafter LAT) is a 400 ha residential development where only 5% of 2 ha lots can be used for construction and the remaining area is medium evergreen forest <30 m in height [23]. LAT is characterized by relatively flat (i.e., non-mountainous) terrain, as most of the Yucatan Peninsula. This site was chosen to evaluate the effects of flight parameters on spider monkey counts (i.e., number of detected individuals) from videos captured during TIR drone flights because a long-term project on wild spider monkeys has been underway there since 2017 [62], and drone flights have been performed in the past [23]. As such, the spider monkeys in LAT are habituated to human presence, are familiar with the noise produced by drone flights, and information on the location of several sleeping sites (i.e., clusters of trees in which the monkeys sleep) is available.

### 2.2. Data Collection Flights

We used a Mavic 2 Enterprise Advance drone to conduct spider monkey surveys. This drone has a high-resolution TIR camera (the lens has a 9 mm focal length, 38 mm for 35 mm equivalent) with an image size of 640 × 512 pixels. This camera has a capture rate of 30 frames per second and a temperature measurement accuracy of 2 degrees (DJI Technology Co, Shenzhen, China). The drone weighs 909 g and has a maximum flight time of 31 min.

We flew over five known sleeping sites of two groups of spider monkeys in LAT (Figure 1; mean distance between sleeping sites: 1052 m; minimum distance: 305 m; maximum distance: 2040 m). Flying over sleeping sites ensures a higher probability to detect and count spider monkeys at the end of the afternoon, at night, or in the early morning than flying over other parts of their home range at any given time, thereby providing better conditions under which to test the drone flight parameters. Little is known in general about spider monkey sleeping behavior after sunset in the wild. In the first nocturnal study on wild spider monkeys, we observed the behavior of the spider monkeys that would arrive and sleep at one particular sleeping site in Los Arboles Tulum for 6–12 h per night (personal observation Denise Spaan and Filippo Aureli). Some nights, single individuals or very small subgroups (e.g., two individuals) would arrive after dark at the sleeping site to join other monkeys that were already there. It is improbable that this occurrence would have substantially influenced the results since flights included all combinations of flight parameters and lasted only approximately 110 s. It is therefore unlikely that subgroup size changed between flights.

We performed 8 drone flights per sleeping site (n = 40). The flight routes were created in Google Earth Pro (version 7.3.4.8248) and flights were performed using the DJI pilot application (version 1.7.0). During each flight, we flew the drone on a straight-line transect (220 m in length) over the sleeping site at a constant speed of 2 m/s four times with different combinations of flight height and camera angle: (1) 50 m high with the camera at −90° (Video S1, Supplementary Materials), (2) 50 m high with the camera at −45° (Video S2, Supplementary Materials), (3) 40 m high with the camera at −90° (Video S3, Supplementary Materials), and (4) 40 m high with the camera at −45° (Video S4, Supplementary Materials). We selected a maximum height of 50 m a.g.l. (i.e., approximately 25 m above canopy level) because we considered it to be the maximum flight height that would ensure sufficient detail in the TIR drone videos to accurately differentiate the spider monkeys from other possible species or sources of noise. We selected a minimum height of 40 m a.g.l. to minimize disturbance to the monkeys by the drone being too close to the tree canopies (i.e., approximately 15 m above canopy level). We recorded one video for each of the 40 flights performed at a flight speed of 2 m/s. We subsequently cut these 40 videos into videos corresponding to the four flight parameter combinations (n = 160 videos) with the VLC program (version 3.0.17.4). We extracted frames from each of the 160 videos using the *Batch Video to Image Extractor* (version 0.1.7) program to simulate flight speeds of 4 and 6 m/s by reducing the number of frames per second from the original video (2 m/s). At the end of this process, we obtained three versions of each of the 160 original videos, corresponding to flight speeds of 2, 4, and 6 m/s, resulting in a total of 480 videos (i.e., 12 videos per flight).

The videos recorded at a camera angle of −45° have a larger field of vision, thereby including sampling areas that are not visible in the corresponding −90° angle videos. Similarly, flights at 50 m above ground level (a.g.l.) produce videos that cover a larger sampling area than videos recorded at 40 m a.g.l. Thus, differences in the number of detected spider monkeys could simply be due to monkeys being present in areas sampled only in one condition, and not due to differential detectability related to the flight parameters. To ensure that the sampled area was the same in all 480 videos, we made the following adjustments. First, to ensure that the sampled area was the same in the videos recorded with the camera angles at −45° and −90° (and therefore that the same monkeys were visible in all transects), we edited the end points of the videos recorded with the camera at −45° to ensure that the 220 m transect started and ended in the same points as in the videos corresponding to the camera at −90°.

**Figure 2:**
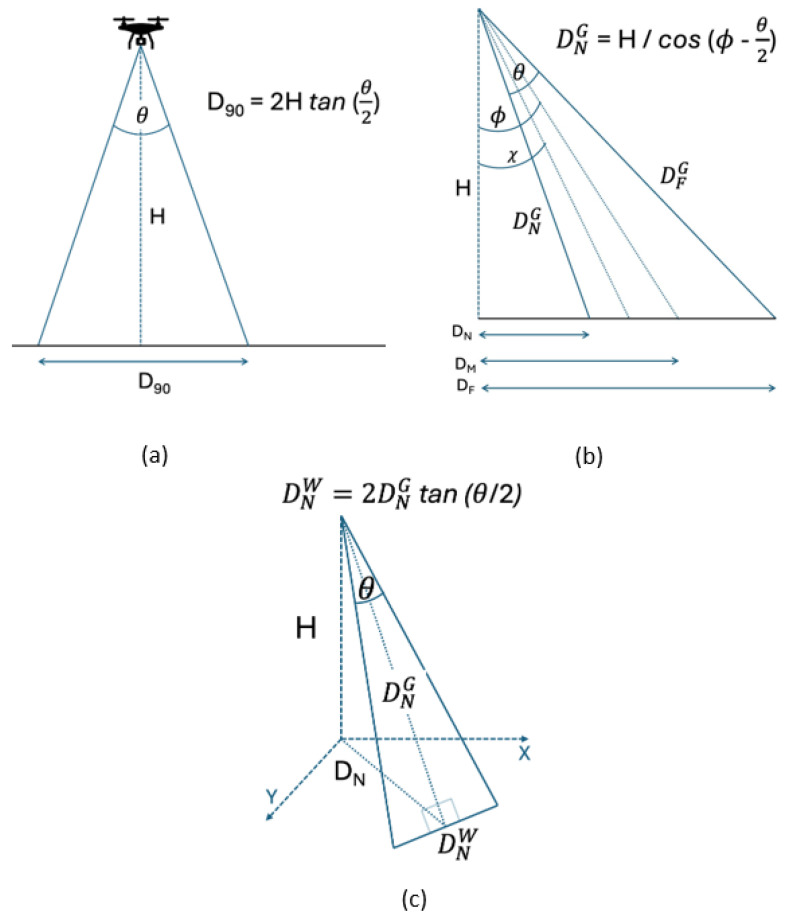
(**a**) Drone at height H with camera pointing directly down (−90°). The value $D_{90}$ is the distance on the ground subtended by a camera with an angular field of view *θ*. (**b**) Side-on view of drone at height H facing toward the right, with the center of the camera field of view pointing an angle of *ϕ*. $D_{N}$ is the distance on the ground from directly below the drone to the nearest point of the drone’s field of view. $D_{N}^{G}$ is the distance from the drone to this point, with G being ground. $D_{M}$ and $D_{F}$ are the distances on the ground from directly below the drone to the middle ($D_{M}$) and farthest ($D_{F}$) point on the drone’s field of view. The angle *χ* is an arbitrary angle between zero and *θ* to generalize the mathematical expressions. (**c**) Reprojected view of (**b**), rotated to show the width (W) of the field of view on the ground at the point nearest to the drone, $D_{N}^{W}$.

To control for the sampling area difference between the two camera angles evaluated (−90° and −45°), we determined how the projected length on the ground of a camera with a field of view *θ* on a drone at height H varies the angle of the camera from pointing straight down, to arbitrary angle *χ*. We first determined the projected distance *D* on the ground when the camera was pointed straight down ($D_{90}$; Figure 2a), and how this varied when the camera was shifted to a different angle ϕ (Figure 2b,c)$,\;D_{N}^{W}$, with *W* being width and referring to the horizontal distance on the ground subtended by the drone camera and *N* being near and referring to the nearest location on the ground in the drone camera field of view. The ratio in the projected distance on the ground is $\frac{D_{N}^{W}}{D_{90}}=\frac{1}{\cos\left(\;\phi \;–\frac{\theta }{2}\right)}$, and for an arbitrary angle, *χ*, the ratio of projected distance is $\frac{D_{\chi }^{W}}{D_{90}}=\frac{1}{\cos\left(\chi \right)}$.

Footage recorded with the camera angle pointed at −45° was 10% wider at the bottom and 56% wider at the top of the image compared to footage taken with a −90° camera angle. We therefore masked 5% on each side of the screen at the bottom and 28% at the top to ensure that the coder counted the number of monkeys in the same sampled area for footage recorded at both camera angles.

Second, to control for the sampling area difference between the two evaluated heights, we used the ground sample distance (GSD; represented by each pixel in an image captured by a drone [63]) to determine that the images recorded at 50 m height were 55.2% larger than those recorded at 40 m height. To review the same area in videos taken at different heights a.g.l., the coder counted the number of spider monkeys observed only in the center of the computer screen by masking 13.8% of the image at each of the four sides of the videos recorded at 50 m a.g.l.

### 2.3. Spider Monkey Detection

**Figure 3:**
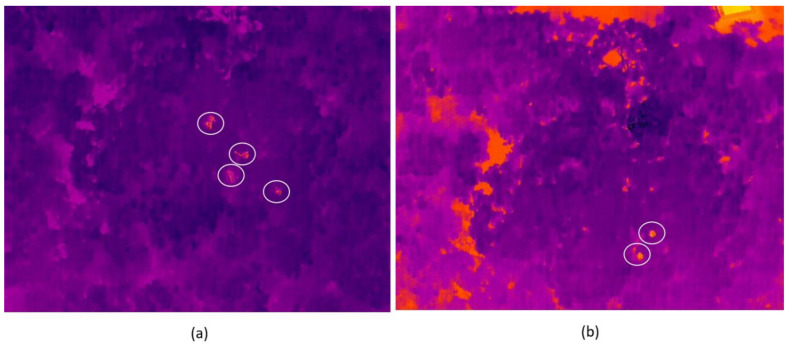
Examples of (**a**) high thermal contrast zones and (**b**) low thermal contrast zones, and how the spider monkeys appear in the videos (inside the white circle).

The 480 videos were coded by Denise Spaan (hereafter the main coder), who has substantial experience in detecting and counting spider monkeys in thermal images and videos. The videos were randomly assigned a unique code; this way, the main coder did not know the flight height and the camera angle of each video prior to its review (i.e., blind coder). We chose this methodology to avoid the main coder biasing toward any of the flight parameter combinations. However, to facilitate data transfer, the videos were grouped according to flight speed, making the coder aware of flight speed ahead of the video review. For each video, the main coder recorded the number of detected spider monkeys. A subset of 120 videos (25%) of varying flight height, speed, and camera angle combinations were reviewed by two additional coders (with a similar level of experience in detecting spider monkeys in thermal videos and knowledge of the morphology and characteristic movements of spider monkeys in the wild) to compare the level of agreement between coders. These two coders did not know the number of monkeys detected by the main coder and were not informed about any of the flight parameters. For each of the 120 videos, the three coders recorded the number of detected spider monkeys in zones of high and low thermal contrast. For each video, the main coder noted the time containing zones of high and low thermal contrast to allow for comparison between coders. High thermal contrast zones were sections of the videos where only forest elements were present and therefore the spider monkeys could easily be distinguished from their surroundings (Figure 3a). Low thermal contrast zones included areas around houses and roads, where the heat from these structures made it more difficult to differentiate spider monkeys from their environment (Figure 3b). All videos were reviewed in VLC Media Player 3.0.8 at normal speed.

### 2.4. Data Analysis

To evaluate whether the three flight parameters were associated with the number of spider monkeys detected in the recorded footage during TIR drone surveys, we ran a generalized linear mixed model (GLMM) with a Poisson distribution, which is appropriate for count data [64]. We ran the GLMM using the *gmler* function in the “*lme4*” package [65] of R version 4.3.0 [66]. We included the number of spider monkeys as the response variable, flight speed (2, 4 and 6 m/s), flight height (40 and 50 m), and camera angle (−45° and −90°) as categorical predictor variables, and flight number as a random intercept to control for the 12 videos obtained from each flight (1 video for each combination of the three flight parameters). We used Cramer’s V coefficient using the *assocstats* function to evaluate potential collinearity between the predictor variables [67] with the *“vcd”* package [68]. Cramer’s V values range from 0 to 1, where 0 refers to a weak or lack of association and 1 refers to a strong association between variables [69]. All comparisons between predictor variables had Cramer’s V values of <0.01, and therefore the three categorical variables were included in the model. By using the function *check_model* of the “*Performance*” package [70], we realized that the model assumptions of normal distribution of residuals, homogeneity of variance, and dispersion of residuals were not met. We dealt with this issue in two ways.

First, we ran a GLMM with the same variables as the initial model with a reduced dataset where we only included the 300 videos of the 25 flights where at least one spider monkey was detected in at least one of the 12 videos corresponding to the combinations of the three parameters of the same flight. That is to say, that if monkeys only appeared in one of the 12 videos from the same flight, all the 12 videos were included in the model. When no monkeys were detected in any of the 12 videos, none of the 12 videos of that flight were included in the model. We included the number of monkeys as the response variable, flight height, flight speed, and camera angle as categorical predictor variables, and flight number as a random intercept in the GLMM. The model met the assumptions of homogeneity of variance and dispersion of the residuals but did not meet the assumption of normal distribution of the residuals.

Second, we ran two separate GLMMs: one for the videos recorded with the camera at a −45° angle (n = 240) and another for the videos recorded with the camera at a −90° (n = 240). In both models, the number of monkeys was included as the response variable, flight height and flight speed as categorical predictor variables, and flight number as a random intercept. Both GLMMs met the assumptions of homogeneity of variance and dispersion of residuals but did not meet the assumption of normal distribution of the residuals. We present the results of the three GLMMs given that the non-normal distribution of the residuals does not affect the model results if the model meets all other assumptions [71,72]. We compared those GLMMs with the corresponding null models, including only the random intercept (flight number), using likelihood ratio tests [73,74].

We evaluated the level of agreement between coders on the number of detected spider monkeys in TIR footage using Fleiss’ kappa coefficient [75]. We used the *kap-pam.fleiss* function in the “*irr*” package [76]. We followed the levels of agreement established in [77]: “poor” (<0.00), “slight” (0.00–0.20), “fair” (0.21–0.40), “moderate” (0.41–0.60), “substantial” (0.61–0.80), or “almost perfect” (>0.81). We compared the number of monkeys counted by the main coder and the two additional coders for the 120 selected videos varying in flight speed, flight height, camera angle, and thermal contrast (Table S1, Supplementary Materials).

## 3. Results

**Figure 4:**
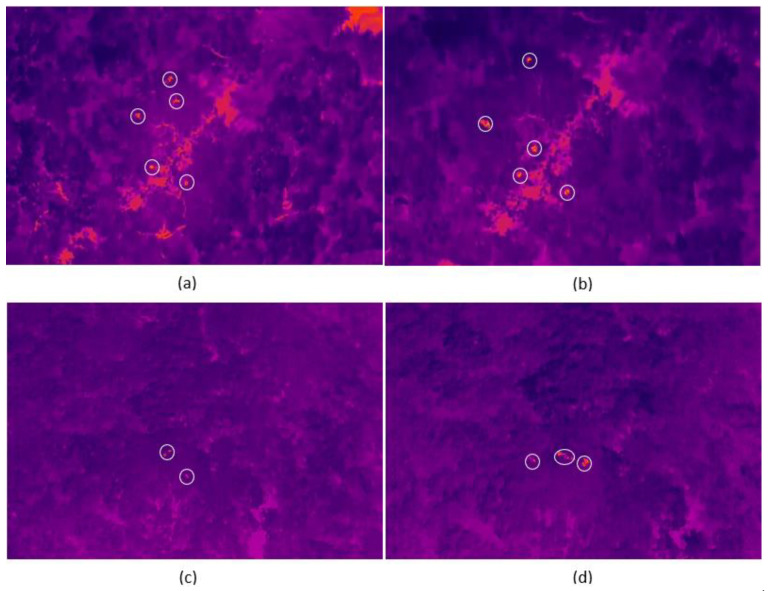
Spider monkeys (within white circles) in TIR drone footage under different combinations of flight height and camera angle: (**a**) 50 m and −90°, (**b**) 40 m and −90°, (**c**) 50 m and −45°, and (**d**) 40 m and −45°.

The main coder detected at least one spider monkey in 263 of the 480 videos (54.7%; Figure 4), with a mean of 2.7 monkeys per video. When the dataset included only videos where at least one spider monkey was detected in at least one of the 12 videos from the same flight (n = 300), the GLMM was not significantly different from the null model (χ^2^ = 2.46, df = 4, *p* = 0.65). Similarly, when the 480-video dataset was divided by camera angle, both GLMMs did not differ significantly from the corresponding null models (videos at −45°: χ^2^ = 1.49, df = 3, *p* = 0.68; videos at −90°: χ^2^ = 1.06, df = 3, *p* = 0.79).

**Figure 5:**
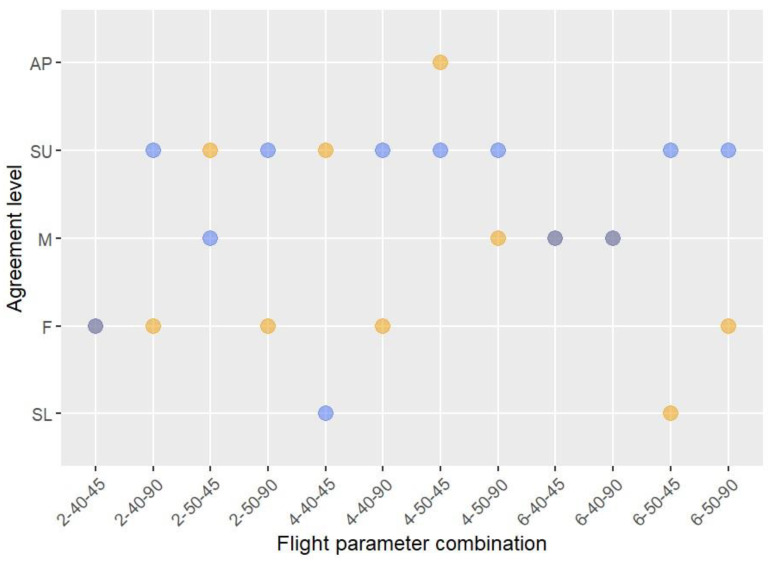
Level of agreement between coders for different flight parameter combinations for high (blue points) and low (orange point) thermal contrast zones. Gray points indicate that both contrast zones had the same level of agreement. The categories of level of agreement between coders on the *y*-axis are as follows: SL (slight), F (fair), M (moderate), SU (substantial), AP (almost perfect). The values of the flight parameter combinations on the *x*-axis are presented in the following order: flight speed (m/s), flight height (m a.g.l.), and camera angle (°).

The level of agreement among coders ranged from “slight” to “substantial” (Figure 5; Table S1, Supplementary Materials). We found that the level of agreement between coders in high thermal contrast zones was substantial for 58% of flight parameter combinations (Figure 5; Table S1, Supplementary Materials). The level of agreement between coders in low thermal contrast zones was “fair” to “moderate” in 75% of the cases, although there was one case of “almost perfect” for the flight parameter combination of 4 m/s speed, at 50 m height and at a −45° angle. In addition, the level of agreement was “substantial” for 83% of the videos at −90°, while for the videos at −45° the level of agreement was “substantial” for only 33% of the cases for high thermal contrast zones. Regarding the level of agreement depending on the speed of flight, we found that, at a speed of 4 m/s, the level of agreement was “substantial” or higher in 62% of the cases, while this percentage decreased for the videos at 2 (37%) and 6 (25%) m/s. For flight height, in general we found that the level of agreement was substantial for 50% of flights performed at 50 m a.g.l., while it was substantial for only 25% of flights performed at 40 m a.g.l.

## 4. Discussion

We evaluated how three flight parameters (flight speed, flight height, and camera angle) influence the number of spider monkeys detected in TIR drone footage. We found no evidence that the selected settings of the three flight parameters affected the number of detected monkeys. When we evaluated the reliability of the results obtained from coding the TIR images, we found, as predicted, the highest levels of agreement between coders for the videos recorded with the camera angle at −90° in high thermal contrast zones. However, contrary to our prediction, we found higher levels of agreement for the intermediate speed of 4 m/s and for flights performed at 50 m a.g.l.

Contrary to our prediction of a higher detection of spider monkeys at the lower speed setting (2 m/s), we found no evidence of flight speed being associated with the number of detected spider monkeys. Our result is similar to that reported for primate species in Costa Rica, although the number of detections in that study was higher at lower speeds for other taxonomic groups (e.g., bats, kinkajous, birds [25]). This taxonomic difference can be explained by the size and social habits of these species because Mesoamerican non-primate arboreal mammals are relatively small and solitary, and thus are likely to go undetected when flying fast. By contrast, spider monkeys are relatively large, live in groups, and gather in subgroups at sleeping sites [78,79], aiding detection in TIR footage. It is also important to consider that, when the drone flies faster, the time a monkey is in the camera’s field of view is less. In real time, some individuals may go undetected. When counts are performed based on post-flight video review, pausing the video to verify spider monkey presence and counts can help ensure detectability for fast flight speeds. We found higher levels of agreement between coders for the videos recorded at 4 m/s than for the videos recorded at 2 and 6 m/s. This indicates that teams with varying degrees of experience in detecting animals in TIR footage can achieve high levels of agreement at intermediate flight speeds. This could be especially useful when automatic detection algorithms are not available and manual coding is performed by people with different levels of experience. Thus, we propose flying at a speed of 4 m/s for TIR drone surveys of spider monkeys as this maximizes flight duration (and thus battery life) while maintaining the same degree of detectability and ensuring high levels of agreement between coders.

We found no evidence of difference in the number of detected spider monkeys between flights performed at 40 and 50 m a.g.l. (equivalent to approximately 15 and 25 m above the canopy). One could argue that this result is due to the better detection expected in footage taken from a shorter distance being countered by the greater likelihood of monkey detection in the larger sampling area footage taken from a higher distance [21]. However, this was not the case as we controlled for the size of the sampling area. Thus, the result may be simply due to the difference between the two flight heights not being sufficiently large to affect the difficulty in detecting spider monkeys [25]. Kays et al. [21] suggested that flights at heights between 80 and 100 m a.g.l. are optimal to balance covering a greater area and maintaining high detectability of arboreal mammals. However, another study carried out in Mesoamerica reported that species could not be identified in >60% of the detections in TIR drone footage recorded at heights between 90 and 100 m a.g.l. [25]. This high percentage of indeterminate detections highlights the importance of selecting a lower flight height, which affords good image quality (to allow for accurate determination of the taxon of interest) and avoids biases in the population estimates obtained from drone flights due to false positives and/or false negatives. Although other mammals similar in size to spider monkeys are rare in LAT, and thus frequent indeterminate detections may not be a problem if flying higher than 50 m, maintaining good image quality is an important factor to consider in other parts of the spider monkey distribution where arboreal mammal species of similar size occur.

To accurately record the temperature of an object with a TIR camera, the object must appear larger in the field of view than a minimum number of pixels [38]. Using Burke et al.’s [38] observation strategy optimization web tool and considering the characteristics of the camera and the spider monkey’s mean body length (0.5 m from the top of the head to the base of the pelvis, without considering the tail [23]), we obtained a maximum distance of 57 m between the drone and the spider monkeys, which would be equivalent to flying at 82 m a.g.l. at our study site. An 82 m flight height is greater than the heights we used and is more in line with flight heights suggested by Kays et al. [21]. However, although it is possible to detect the temperature of individuals at this height, the level of detail of the TIR footage is low, which would make it difficult to correctly identify the species of interest. We noticed a similar pattern when reviewing our TIR footage, as the level of detail was greater in videos from flights performed at 40 m height than in videos from flights performed at 50 m height. Therefore, it is unlikely we would have been able to determine with certainty that heat signatures were spider monkeys if flying at a height of 82 m a.g.l. To solve this issue, other researchers have proposed using a visual camera (RGB) in addition to a TIR camera to aid in species identification [21], particularly if flights are performed just after sunrise when the TIR camera can still pick up strong heat signatures. This can be very important in sites where the presence of other species with similar characteristics can make the identification of individuals difficult and can produce biased estimates. Based on our results and experience, we recommend flying the TIR drone at a height of 40 m a.g.l. on flat ground such as that of LAT. Although the topography across the distribution of the Geoffroy’s spider monkeys is highly variable, we recommend flying at a maximum height of 15 m above the canopy to maintain a good level of detail in the TIR footage, allowing for the correct taxonomic identification of the detected individuals, even though this implies longer flight times and higher battery consumption to cover the same area.

Despite the importance of the camera angle for the field of view [38,80], only few studies have evaluated its influence on animal detectability in footage obtained from drone surveys [38,45]. We found no difference in the number of spider monkeys detected in the same sampling area between flights with camera angles at −45° and −90°. Our results differ from those previously reported [35,45]. For example, a −90° angle improved the detection of potential “poachers” in forest canopy gaps compared to a −45° angle when using TIR drones [35]. By contrast, the probability of “poacher” detection was higher at a −45° angle when an RGB camera was used [35]. Similarly, in an experimental design where decoys of different species varying in shape and size were used to assess detectability, the number of incorrectly identified decoys was lower with a −45° angle compared to a −90° angle when using an RGB camera in open areas dominated by grasslands and shrubs [43]. These results with RGB drones may be due to the fact that more information about the size and/or color of the individuals can be obtained at a −45° angle than at a −90° angle. However, this information is not as relevant when using a TIR camera.

Features such as tree trunks and branches are more visible in the −45° angle videos, making it more difficult to differentiate between them and the monkeys, especially when the monkeys do not move, as these features retain heat and therefore appear as bright as the monkeys in TIR drone footage. Additionally, when using a camera angle of −45°, the apparent size of the objects varies across the field of view due to the image distortion produced by this camera angle [38], which increases the difficulty of detecting spider monkeys. The high level of agreement between coders in TIR footage taken with the camera at a −90° angle supports the ease of detecting monkeys with this setting. We therefore recommend performing spider monkey TIR drone surveys with the camera angle at −90°, although this implies longer flights and higher drone battery consumption to cover the same area than if the flights were performed at a −45° angle.

Even though not all coders had the same level of experience in detecting spider monkeys in TIR footage, agreement between coders was high. This result is promising because it indicates that individuals with prior knowledge of the species of interest (e.g., its morphology or types of movement) may be able to appropriately code videos despite having little to no previous experience in detection in TIR footage, in line with findings reported in previous drone studies where manual video processing was performed [20,23,25,81]. In some studies, a higher level of agreement among coders was found with increased familiarity with the study species in addition to having previous experience working with images or videos recorded with drones [23,81], indicating that coder agreement likely improves with training. We therefore recommend training coders in TIR footage processing to familiarize themselves with the appearance and characteristics of the species of interest before starting coding, especially if more than one coder manually reviews the TIR footage.

The level of agreement between coders was likely influenced by the characteristics of the study site. In Los Arboles Tulum, the forest is interspersed with houses and dirt roads that absorb solar radiation and the spider monkeys tend to sleep in trees close to the houses. It was therefore difficult at times to differentiate the spider monkeys from these heat-absorbing elements because they masked the heat signature of the spider monkeys. This is reflected in the higher levels of agreement between coders for high thermal contrast zones than for low thermal contrast zones. We therefore recommend that future studies consider thermal contrast within their study area and include this variation in analyses, when possible, to avoid biases when manually processing TIR drone footage with multiple coders.

The high consistency in the number of spider monkeys detected in high thermal contrast zones (i.e., forested areas) between different observers is promising for future TIR drone surveys, as surveys are urgently needed across large portions of the spider monkey distribution, most of which is still forested to some degree [58]. A high level of inter-coder agreement implies that teams of coders can be involved in manual data processing, speeding up data gathering, when access to machine learning algorithms is not available. As such, drone TIR surveys with manual processing of video footage are a potentially rapid survey technique in forested areas, which can aid to understand the current state of spider monkey populations.

Although our study represents a step forward toward understanding the implications of flight parameters for counting spider monkeys with TIR drones, it is important to consider the following aspects. First, we did not know the actual number of spider monkeys that were present at the sleeping site during the flights. This implies that we could not know whether any combination of the three flight parameters allowed us to count the exact number of monkeys that were present. Still, it is feasible to assume that the majority of individuals present were detected based on a previous study at the same study site, in which the number of individuals counted in the TIR drone images was similar to ground counts when spider monkey subgroups were small, and even higher numbers of individuals were detected from the TIR images when subgroups were large [23]. Second, it is possible that, during or after some of the four transects of the same flight route, some spider monkeys may have moved to/from areas outside the drone’s field of vision or to/from the middle strata of the canopy, making the number of detectable individuals different between transects. To minimize the occurrence of this situation, we performed the flights at sleeping sites, where spider monkeys are less likely to move. Third, a combination of the flight parameters could have affected the behavior of the monkeys, which could have altered their detection (e.g., they moved away from the canopy). While this is a possibility, we believe that this was unlikely during our study because these monkeys have previously been exposed to drone flights [20,23], to which the spider monkeys did not respond with substantial behavioral changes [23]. It is therefore reasonable to assume that the monkeys did not descend to the lower levels of the canopy in response to the drone. Fourth, coders may have mistaken an individual of another species for a spider monkey. However, this was likely a rare event in our study as the only other arboreal animal of similar size at LAT is the black howler monkey (*Alouatta pigra*), which is rare in the region and [82] was reported on fewer than 20 occasions during more than 5 years of monitoring in LAT (personal observation by Denise Spaan and Filippo Aureli). Fifth, we conducted the flights using a Mavic 2 Enterprise Advanced drone, which is a relatively small model and does not produce a significant amount of noise. The level of disturbance generated is therefore expected to be minor and to not negatively influence spider monkey behavior [83]. However, it is possible that larger drone models that generate more noise may cause a greater behavioral response from the spider monkeys. Hence, when planning to monitor animal populations with drones, it is important to consider these aspects in the study design to define the optimal flying altitude and speed [83].

Evaluating the influence of different flight parameters on the number of individuals detected in footage recorded during TIR drone flights is an essential first step to develop optimal and standardized flight protocols for TIR drone surveys of arboreal mammals. Our study contributes to the standardization of such protocols, with which it is then possible to obtain reliable data on the distribution and abundance of species in locations that are difficult to access or where the species have not been studied. Knowing the distribution and abundance of different species is critical to understand the current status of their populations and propose effective and informed actions for their conservation [84].

## 5. Conclusions

This study provides important insights toward the optimization of flight parameters for spider monkey detection using TIR drones. Although we found no evidence that flight speed, height, or camera angle significantly affect the number of monkeys detected, our results suggest that an intermediate speed of 4 m/s and a camera angle of −90° provide higher levels of agreement among coders, regardless of their level of experience. This is especially relevant when automatic detection algorithms are not available, and analysis relies on manual coding. In addition, flying at a height of 40 m a.g.l. (approximately at 15 m above the canopy) is optimal for maintaining a good level of detail in the TIR drone footage to ensure accurate spider monkey identification, especially in areas with high thermal contrast. These findings are crucial for the standardization of monitoring protocols, facilitating studies on arboreal mammal distribution and abundance, which is essential for the conservation of spider monkeys and other arboreal mammals. 

## Figures and Tables

**Table 1:** Flight parameters and camera types used in studies of primates conducted with drones.

Species	Camera Type	Flight Height Above Ground Level (m)	Drone Speed (m/s)	Camera Angle (°)	Reference
***Alouatta palliata***	TIR and RGB	80–100	3	N.S.	[21]
	TIR	90–100	2.8–5	−90	[25]
***Ateles geoffroyi***	TIR and RGB	80–100	3	N.S.	[21]
	TIR	90–100	2.8–5	−90	[25]
	TIR	60–70	N.S.	−90	[23]
	RGB	35–40	0.8	−90	[20]
***Hylobates moloch***	TIR and RGB	10–120	5	−90	[42]
***Macaca fascicularis***	TIR	10–100	8.5–11	N.S.	[30]
* ***Macaca fuscata***	TIR and RGB	120–150	2–5	N.S.	[36]
***Nasalis larvatus***	TIR	80–120	1–7	−90	[29]
* Nomascus gabriellae	TIR and RGB	50–80	N.S.	N.S.	[47]
***Nomascus hainanus***	TIR	50	N.S.	N.S.	[24]
	TIR and RGB	5–50	5	−90	[33]
***Nomascus nasutus***	TIR and RGB	30–120	**	N.S.	[48]
***Pan troglodytes***	RGB	120	N.S.	N.S.	[14]
	TIR	N.S.	N.S.	−90	[49]
***Pan paniscus***	TIR	100–120	8–12	N.S:	[50]
**^#^** ***Papio anubis***	RGB	20	N.S.	N.S.	[51]
***Pongo* sp.**	TIR	60–200	N.S.	N.S.	[46]
***Pongo pygmaeus***	TIR	80–120	1–7	−90	[29]
***Presbytis comate***	TIR and RGB	10–120	5	−90	[42]
***Propithecus tattersalli***	RGB	15–55	N.S.	N.S.	[52]
* Pygathrix cinérea	TIR and RGB	50–80	N.S.	N.S.	[47]
***Rhinopithecus*** ***roxellana***	TIR and RGB	150–250	6	−90	[53]
***Trachypithecus auratus***	TIR and RGB	10–120	5	−90	[42]
	TIR and RGB	20–100	8.5–11	N.S.	[54]
* Trachypithecus delacouri * Trachypithecus hatinhensis	TIR and RGB	50–80	N.S.	N.S.	[47]

* This study was performed with captive primates in enclosures. ** Drone speed was not standardized. ^#^ It is a preprint and has not yet undergone peer review. N.S. = Not specified.

## Data Availability

The original data presented in the study are openly available in FigShare at [https://doi.org/10.6084/m9.figshare.26359423] accessed on 23 July 2024.

## Supplementary Materials

The following supporting information can be downloaded at: https://www.mdpi.com/article/10.3390/s24175659/s1, Table S1: Level of agreement for each of the different flight parameters combinations, for each of the thermal-contrast zones. We present the number of subjects evaluated for each category, the number of coders, the Kappa and *p*-value, and the corresponding agreement level; Video S1: Route of the drone flight at 50 m high with the camera at −90°; Video S2: Route of the drone flight at 50 m high with the camera at −45°; Video S3: Route of the drone flight at 40 m high with the camera at −90°; Video S4: Route of the drone flight at 40 m high with the camera at −45°.
