# Supplementary material for: Evaluating Thermal Infrared Drone Flight Parameters on Spider Monkey Detection in Tropical Forests
Source: Sensors (Basel). 2024 Aug 30;24(17):5659. doi: 10.3390/s24175659 (PMC11397880; doi:10.3390/s24175659)
Supplement: Supplementary file 1 [file sensors-24-05659-s001.zip › Suplemmentary materials-15-07-2024.pdf]

Supplementary Materials

Table S1: Level of agreement for each of the different flight parameters combinations, for each of the thermal-contrast zones. We present the number of subjects evaluated for each category, the number of coders, the Kappa and p-value, and the corresponding agreement level.

**Flight parameter combination**	**Thermal contrast zone**	**Subjects**	**Raters**	**Kappa**	**z**	**p-value**	**Agreement Level**
**2-40-45**	Low	5	3	0.28	2.57	>0.01	fair
**2-40-45**	High	10	3	0.278	2.99	>0.01	fair
**2-40-90**	Low	9	3	0.402	4.28	>0.01	fair
**2-40-90**	High	10	3	0.633	4.49	>0.01	substantial
**2-50-45**	Low	7	3	0.747	7.01	>0.01	substantial
**2-50-45**	High	10	3	0.528	4.92	>0.01	moderate
**2-50-90**	Low	8	3	0.294	2.97	>0.01	fair
**2-50-90**	High	9	3	0.665	7.46	>0.01	substantial
**4-40-45**	Low	7	3	0.69	6.1	>0.01	substantial
**4-40-45**	High	10	3	0.163	1.35	0.177	slight
**4-40-90**	Low	8	3	0.306	2.38	>0.05	fair
**4-40-90**	High	10	3	0.607	8.16	>0.01	substantial
**4-50-45**	Low	8	3	0.854	6.92	>0.01	almost perfect
**4-50-45**	High	10	3	0.618	5.29	>0.01	substantial
**4-50-90**	Low	9	3	0.427	4.27	>0.01	moderate
**4-50-90**	High	10	3	0.746	6.67	>0.01	substantial
**6-40-45**	Low	7	3	0.51	6.25	>0.01	moderate
**6-40-45**	High	8	3	0.56	5.64	>0.01	moderate
**6-40-90**	Low	8	3	0.489	4.25	>0.01	moderate
**6-40-90**	High	10	3	0.602	5.29	>0.01	moderate
**6-50-45**	Low	6	3	0.173	1.1	0.272	slight
**6-50-45**	High	9	3	0.773	7.66	>0.01	substantial
**6-50-90**	Low	8	3	0.364	2.91	>0.01	fair
**6-50-90**	High	10	3	0.637	6.6	>0.01	substantial

Video S1. Route of the drone flight at 50 m high with the camera at -90°.

Video S2. Route of the drone flight at 50 m high with the camera at -45°.

Video S3. Route of the drone flight at 40 m high with the camera at -90°.

Video S4, Route of the drone flight at 40 m high with the camera at -45°.
